# Supplementary material for: Content-rich biological network constructed by mining PubMed abstracts
Source: BMC Bioinformatics. 2004 Oct 8;5:147. doi: 10.1186/1471-2105-5-147 (PMC528731; doi:10.1186/1471-2105-5-147)
Supplement: Additional File 5 — The original Chilibot query results of the term "long-term potentiation (LTP)" and 22 other terms, limiting the latest references analyzed to the years 1990, 1995, 2000, and 2004. [file 1471-2105-5-147-S5.bz2 › chilibotAdditionalFile5/ltp2000/html/PLC_PKA.html]

 


 **PLC** and **PKA** 
  
Found 59 abstracts in PubMed,  **30 abstracts were retrieved and analyzed**.  


---

 Search Google  |
 PDF files only 
|  EDU domain only 

---

**Interactive relationship** (e.g. stimulation, inhibition, etc)

- The resulting  **PKA**  activation phosphorylates PLCbeta3 and appears to block the ability of Gbetagamma to activate  **PLC** .  Ref: 9556582 J Biol Chem, 1998
- A combined administration of SQ 22536 AC inhibitor followed by U 73122  **PLC**  inhibitor, or U 73122 followed by H 89  **PKA**  inhibitor, virtually abolished the response to vasopressin.  Ref: 9824711 J Physiol, 1998
- Similarly,  **PKA**  activation with Sp cAMP or forskolin had no effect on  **PLC**  stimulation by carbachol.  Ref: 10353692 Cell Signal, 1999
- We suggest that oxytocin activates two distinct signalling pathways in vagal neurons one which is cAMP dependent, but  **PKA**  independent, and one, unidentified, which is  **PLC**  beta and cAMP independent.  Ref: 9517466 Eur J Neurosci, 1997
- Inhibition of  **PKA**  with H89 or Rp cAMP did not alter the ability of carbachol to activate  **PLC** .  Ref: 10353692 Cell Signal, 1999
- in contrast,  **PLC**  inhibitor U 73122 did suppress adhesion, suggesting the possibility that  **PLC** ... PKC, or  **PKA** , may be involved in this process.  Ref: 9682000 Cell Immunol, 1998
- The suggested mechanism is based on the regulation of local calcium release by cyclic AMP dependent protein kinase  **PKA**  and relies upon two experimental observations first, some G protein coupled signaling pathways activate  **PLC**  and regulate adenylate cyclase at the same time, leading to IP3 production and altering  **PKA**  activity via changes in cAMP level.  Ref: 10378083 Cell Calcium, 1999

- :-)

  **Parallel relationship** (e.g. studied together, co-existance, homology, etc.)

  - Desensitization of  **PLC**  was unaffected by  **PKA**  blockade, partially mimicked by phorbol ester, and not reproduced by agents that selectively activated AC.  Ref: 9277393 Am J Physiol, 1997
  - Furthermore, elevation of cAMP may inhibit IP3 production and calcium i mobilization through mechanisms involving  **PKA**  dependent phosphorylation of  **PLC** , G proteins, IP3 receptor and or IP3 metabolizing enzymes.  Ref: 9376222 Cell Signal, 1997
  - To screen the possible mechanisms involved in cell volume regulation in these cells, we examine intracellular mechanisms and second messengers listed as follows phospholipase C  **PLC** , phospholipase A2 PLA2, tyrosine kinase TK, protein kinase C PKC, protein kinase A  **PKA** , and cAMP.  Ref: 9481486 Cell Signal, 1998
  - The data suggest that mammalian insulin sensitive cells and yeast share part of the key regulatory mechanism consisting of PP2A,  **PKA** , cAMP PDE, and GPI  **PLC**  involved in the transduction of the insulin signal from the respective receptor systems to glycogen synthase and phosphorylase.  Ref: 10684630 Biochemistry, 2000
  - The insulin concentrations effective in modulating PP2A,  **PKA** , cAMP PDE, and GPI  **PLC**  activities correlate well with those required for half saturation of the specific binding sites as well as for stimulation of protein phosphorylation and glycogen accumulation.  Ref: 10684630 Biochemistry, 2000
  - In this report, we present the first study of the  **pKa**  values of histidines of a PI  **PLC** .  Ref: 9300493 Protein Sci, 1997
  - We also evaluated the relative contributions of extra and intracellular calcium sources and of cAMP dependent protein kinase  **PKA**  and phospholipase C  **PLC** .  Ref: 9924628 Cell Calcium, 1998
  - These results suggest a function for PKC and  **PKA**  in M3 stimulation of arachidonic acid release and calcium mobilisation but not in  **PLC**  activation.  Ref: 10353692 Cell Signal, 1999
  - Analyses on inhibition and phosphorylation of signaling molecules suggested that GH induced lipolysis stimulation is dependent on gene expression and not mediated through  **PKA** , PKC, PLA,  **PLC** , nor MAPK pathway but possibly through JAK STATs pathway.  Ref: 10854705 Mol Cell Endocrinol, 2000
